# Innovation capabilities in the convergence trend of higher education from the perspective of quality management

**DOI:** 10.3389/fpsyg.2022.979059

**Published:** 2022-08-24

**Authors:** Jingjing Wu, Yixian Gu

**Affiliations:** ^1^School of Electronic and Information Engineering, Changshu Institute of Technology, Suzhou, China; ^2^School of Business, Changshu Institute of Technology, Suzhou, China

**Keywords:** innovation capabilities, quality management, admission process, placement process, higher education institutes

## Abstract

With changing trends and technology, the education system has evolved from a traditional to a modernized, qualitative, and innovatively sustained education system. Many factors contribute to process innovation and quality management benchmarks. This study has two primary goals: (1) determining the causal relationship between TQM and innovation capability, and (2) determining whether the exam, admission, and placement process have any effect on TQM and identifying whether TQM can act as a mediator between the admission, exam, and placement process and innovation capabilities. Furthermore, the study used TQM in multiple dimensions (quality management and leadership, staff interaction, institute productivity, and control and measurement of processes). As a result, the current study is the only one to look at TQM with its specific dimensions as a mediator, specifically in higher education. The survey and correlational methods were chosen to test the theoretical framework established using resource-based theory and explicitly based on structural equation modeling using Partial Least Square. A structured questionnaire based on a five-point Likert scale was also distributed to 350 professors (faculty members) from Chinese universities to assess the research constructs. The findings revealed that TQM positively and significantly impacts innovation capabilities. Besides, the admission, exam, and placement process is inextricably linked to TQM’s dimensions and innovation capabilities. TQM also mediated significantly, and all hypotheses tested supported the findings. Future researchers should look into collaborative innovation capabilities and compare teachers’ innovation capabilities in higher education, according to the study.

## Introduction

With advances in technology and global knowledge and skill, the education sector is striving to upgrade itself by adding new technological, theoretical, and practical knowledge to the fields. The advancement of knowledge and educational standards in all fields has created a competitive environment that every educational institute must strive for. To gain a competitive advantage, institutes must develop their innovative capabilities and improve the quality management of their institute in all aspects (Abbas, 2020). After the pandemic break, there are several fundamentals that, aside from quality education, require advancements and process innovation. Post-pandemic technological advancements have been dominating and widely known. Admission, exam, and placement processes were a few other aspects of the education sector that were highlighted for quality management and developing innovative capabilities. Because of the complexities encountered by students during online classes and enrollment in higher education institutes, the need to work on these areas were emphasized.

Innovative capabilities in education define the future of knowledge advancements and an institute’s ability to succeed in developing an enhanced knowledge gateway. There is a greater emphasis on innovation development in an institute with skill development and knowledge development. Another factor that emphasizes the innovative up-dating of educational institutes is technological advancement. The emphasis on higher education institutes’ innovation capacities is increasing because high-quality educational services can improve skill development and ultimately help students improve their educational performances, learn new skills, and be satisfied with the education system. Even though China has many higher education institutions, quality is the only criterion by which an institute or service firm such as an educational institute can be recognized (Kim et al., 2022). Higher education institutes are founded not only on quality education but also on quality services that can help the institute establish a standard. It is necessary to develop quality services by innovating existing services to build and maintain those standards that can provide more convenience to students. Quality education is not the only thing that students expect from educational institutions; they also expect some extra conveniences and services that can be developed through innovative strategies. The development of innovative institutional capabilities is the primary factor that defines the institute’s future goals. Successful institute sustainability can be ensured as long as an innovative capacity formation is sustained.

Higher education institutions are working hard to attract high-quality human resources in this regard (Dyer, 1970). As a result, more robust mechanisms and quality controls are needed to meet the institution’s quality improvement goals. In this regard, government agencies can help academic research and innovation by providing high-quality services (Lee, 2022). Students are becoming more aware of the high-quality educational services offered by universities. Higher education institutions worldwide are implementing higher quality services to satisfy students and improve their innovative capabilities. They compete for high-quality education services on a global scale. Service quality is the most crucial factor in improving educational quality in these competing countries to sustain innovation (Steppacher et al., 2021; Kim et al., 2022). Previous literature studies have discovered a positive relationship between service quality and innovative capabilities (Mulay and Khanna, 2020; Iqbal et al., 2021; Steppacher et al., 2021; Kim et al., 2022). Nonetheless, the need for quality improvement through innovative capabilities in higher education has dominated, as a single component cannot provide improved quality services. Aside from innovative capabilities, educational stakeholders such as students, faculty, workers, and government ruling bodies must contribute to an institute in their designated roles. Global educational institutions have recognized the importance of service quality and are concerned about its smooth provision and utilization (Steppacher et al., 2021). Although service quality has a positive effect on innovative capability, the reason for how and why service quality affects innovative capability has yet to be investigated (Abbas, 2020).

Some prerequisites must also be reached before implementing innovative capabilities. The innovative capabilities of an institute can only be advanced through awareness, information seeking, and the application of new approaches, which will allow them to gain practical experience and become experts in improving the organization’s operational process with novelty (Mulay and Khanna, 2020; Lee, 2022). Another issue with innovative capabilities is that not all organizations have the structure and processes to use them, so they may have to suffer the consequences to effectively gain a competitive advantage (Arrieta and Avolio, 2020). At the same time, innovative capabilities for improved service quality and a relevant institutional structure must be implemented to gain adhesion. The quality management standards must be synchronized in all the institute’s processes. The principal highlighted processes included in this study are admission, examination, and placement. The terms “administrative quality” and “administrative processes” match some of the authors’ response factors. These factors attract international and local students to be a part of a standard quality institute delivering all these facilities on the campus. Despite providing all these infrastructures, a good and well-thought admission process is required to meet quality management standards and satisfy prospects (Agung Nugroho et al., 2020; Prameswari et al., 2020). Besides the other primary process, the examination process, the education system across China is managed by the Ministry of Education, a mostly government-run system. In the given system, exams are the baseline for measuring students’ academic performance. Likewise, as soon as pupils begin school in China, they are subjected to several exams (Qi, 2004).

Today’s education system is evaluated based on a variety of factors, including the quality of the examination process and the quality of exam-based evaluation processes. Students’ satisfaction with the exam process in quality management can be measured in various ways, including the quality of an institution’s entrance exam, course evaluation exams, exam administration procedures, and exam evaluation based on student learning. As a result of COVID, the exam process was transformed from traditional to online. Moreover, now, due to the switch to the traditional examination system, there are problems with quality management. Exam satisfaction and fairness are viewed differently by professors and students. It is expected that the digitization of education will continue beyond the control of COVID, necessitating the need to manage the process by introducing some innovative solutions. Innovative solutions should not only replace the traditional examination system but also meet the technological development needs of the postsecondary education system.

The admission process in educational institutes is another indicator of quality management in the education sector. The educational system’s current admissions process has both advantages and disadvantages. Admission flaws vary according to educational institution and culture. As a result of the admissions process, many capable students are denied access to quality education. The social structure to which students belong is the first issue to be addressed, followed by the education ranking system. An institution’s admission test and selection criteria may be based on social life standards, which may jeopardize educational affordability. Direct admission to higher education, with or without an entrance exam, is another issue. Direct admission with the clearance of a language test as part of the entrance exam is one option, whereas admission to an international university for higher education without an entrance exam is another. Without an entrance exam, the student must first complete a foundation year at the host university before being admitted to the university for higher education. The admissions process for online learning programs has complicated educational quality. Students must learn, but their fear of poor admissions quality prevents them from doing so. To deal with such massive complexities, technologically integrated institutes should devise novel strategies capable of handling both online and physical enrollment procedures while also adapting to future unusual circumstances.

The higher education placement process is intended to place students with relevant skills and abilities in their respective job firms. Companies participating in job fair programs may use face-to-face selection in the placement process. Placement portals designed for students are also used in an institute’s placement process, and University placement portals serve as a virtual meeting ground for companies and job-seeking students. Due to the traditional selection process, placement programs in educational institutes have become obsolete. The placement process in educational institutes has ceased and become obsolete during and after the COVID era. One reason could be the need to upgrade online placement programs as a post-COVID placement solution. In addition, the educational system has not been upgraded to the point where it can produce skilled candidates for jobs that companies can consider hiring through institute placement programs. University placement programs must upgrade and innovate in light of current environmental and educational conditions. In terms of providing student satisfaction, the university’s quality management through its placement program is becoming a concern. Quality control in the institute placement process necessitates some creativity. Management of innovation in the placement process is analogous to skill management in educational institutions. The placement programs can be sustained if the institute’s educational quality ensures talented students and graduates. Furthermore, with the ever-rising need and interest of aspirants in higher education, Government of China is making use of several examination processes as a measure of gauging students learning outcomes and the intensity of quality of education they have received (Hill et al., 2010). Moreover, there is a significant focus on the placement process and professional skills to improve educational results in higher education practice and legislation (Mustafa et al., 2020). University-based programs are often considered adequate in preparing students for a seamless transition into the workforce. Another element included in the knowledge curriculum for students in higher education is practice-based learning, which exposes students to real-world situations and develops their practical knowledge, both of which improve the quality of education (O’Leary et al., 2022). Industrial knowledge and job skills are critical components of teachers’ competitiveness growth (Zhang et al., 2018). However, the past literature fails to understand the exam, admission, and placement process on innovative capabilities through four quality management processes. Therefore, the current study intends to examine the mediating role of quality management dimensions in bringing innovative capabilities.

Literature indicates that many studies have examined the role of innovative capabilities and quality orientation on the performance of higher education institutions (Prameswari et al., 2020; Iqbal et al., 2021; Steppacher et al., 2021; Kim et al., 2022). For instance, researchers have emphasized, i.e., thoughtful institutes design (Mulay and Khanna, 2020), teacher involvement (Prameswari et al., 2020), and the performance of the teaching-learning procedure (Caspersen and Smeby, 2021). However, there is research on how to increase the quality of the admissions or placement process (Iqbal et al., 2021; Steppacher et al., 2021; Engdaw, 2022; Lee, 2022). Likewise, students’ satisfaction is another determinant in institution quality improvement, and actions must be carried out accordingly (Alotaibi et al., 2016; Kim et al., 2022). Consequently, the interaction between admissions, exams, and placement and its impact on quality leading to innovative capability, is a fundamental gap that has not been explored yet. The need for innovation in higher learning institutions is on the rise, as indicated by the (Kaputa et al., 2022) study, which highlighted that social innovation in higher education was a sudden step toward quality improvement. However, there is still a gap in education service quality that needs to be examined and improved. According to (Nurmamatovich, 2022), there is a need to implement innovative changes to education culture in higher education institutes through the process of upgrading technological capabilities, which will eventually help improve the quality and effectiveness of the education process. Higher education institutes need value co-creation by incorporating innovation into the education process, eventually transforming HR operations, educational services, process design, and educational service delivery (Cavallone et al., 2022). With global digital transformation, there is also a need to transform the education sector, bringing innovation through the digital transformation that will bring novelty to higher education management strategy and administrative process and provide stability to the student experience and expectations (Mohamed Hashim et al., 2022).

The education sector is constantly changing, especially in the admission examination process, where students perceive a need for innovative policy to establish a more convenient and high-quality admission exam process (Mohamed Hashim et al., 2022). The pandemic has transformed the traditional examination process into a more complex and digitized environment that should be innovated rather than reverted with proper strategy formulation (Assiri et al., 2022). It is necessary to improve the quality of the examination process in universities to meet students’ desires for a quality-assured education system, which can result in quality-assured exam processes in institutions (Avramkova et al., 2021). Higher education institutes’ admission processes are improving by providing equal access to students from all regions, but there is still room for improvement in the technical complexities of the admission process, which necessitates some upgrading and process innovation (Yetra and Hakim, 2022). Many educational institutions have implemented a placement process, but a gap in placement process management and development has created a problem for students’ employability (Ngonda et al., 2022). The online placement process created technical language barriers and information security issues during COVID, and it is critical to improving quality management by providing a satisfying placement process that resolves the students’ technical issues (Ha, 2022). Changing trends in the educational and professional communities necessitate some anticipated changes to institute-organized placement programs (Jung, 2022), and it is thus imperative to innovate the placement process following changing professional and knowledge trends. As a result, this study focuses on selected universities to discover and quantify quality in the procedure, thereby increasing the educational institution’s quality. The findings of this study should help institutions identify and prioritize actions that improve institutional quality and increase innovation capabilities. Top universities worldwide are concerned about management education quality assurance (Sopa et al., 2020; Lee, 2022). With changing trends and educational development, the higher education sector must constantly update and innovate to meet quality education standards. Higher education is essential for developing a country’s capabilities and laying the groundwork for its prosperity. The current study adds to the literature on innovation capabilities in higher education convergence trends from the standpoint of quality management. The researcher has highlighted the fundamentals of education management in this study, which can build a standard of innovation and quality management by becoming proactive in addressing students’ fundamental issues.

The study’s findings will help higher education institutes develop their innovative capabilities by strategizing their exam, admission, and placement policies to accommodate both online and physically enrolled students. This research will be an initiative to develop a transformed education and career facilitation for students and faculty and institute management. The institutional processes are being transformed as the education system is now integrated with online and physical classes. This study will provide an opportunity for institutes to receive feedback and improve their enrollment in the placement process. Finally, this research will help university teachers implement quality management standards to improve their ability to provide quality services. This paper is divided into five sections: the first is an introduction, the second is a literature review, the third is about methodology, how and where to collect data, the fourth is about detailed data interpretation, and the fifth is about the discussion. The final section discusses the study’s implications, future recommendations, and conclusion.

## Literature review and hypotheses development

**FIGURE 1:**
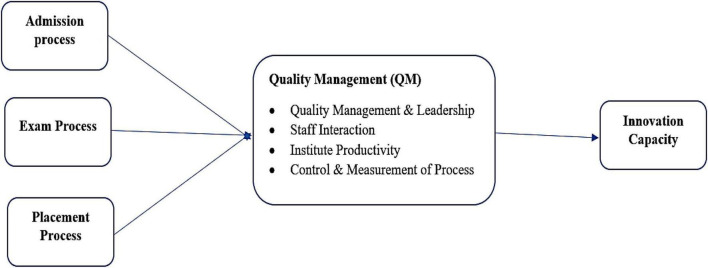
Theoretical framework.

The theoretical framework for this study is critically developed by analyzing previous literature. In this regard, the literature on the quality management process concerning innovation capabilities was examined to identify the significant contributing area. In this regard, it was discovered that few studies addressed the role of the admission, exam, and placement processes on quality management leading to innovation capabilities, particularly in higher education institutes. Furthermore, this theoretical framework focuses on the quality management process’s mediating role in leadership, staff interaction, institute productivity, control, and process measurement. The theoretical framework of the research is available in Figure 1.

### Admission process and innovation capabilities in higher education through quality management

Quality management across higher education is a dynamic process constantly evolving with ever-changing student expectations and quality standards for faculty and staff. For the successful adoption of Quality management in higher education, institutions and faculty must take proactive actions. Likewise, HEIs are supposed to cope with the prevailing quality-related development at both micro and macro environmental levels, despite having certain socio-political and socio-economic constraints (Camilleri, 2019). In the admission of new students, a new mechanism for quality improvement has been implemented: the zoning system. It is expected to assure objective, accountable, transparent, and non-discriminatory admittance of new students to facilitate increased access to education services. Because without prejudice and to provide equal opportunity for all students to receive a formal education, regardless of cognitive or economic capacities (Ibrahim et al., 2021). However, due to the mindset of some parties, there are still roadblocks to implementing total quality management (Arrieta and Avolio, 2020).

Teachers and administrators evaluate, design, implement, and measure quality (Prameswari et al., 2020). China’s education system is shifting toward more technology-based student-centric quality management approaches. Therefore, an honest, attentive approach to learners’ needs is encouraged and crucial to student-staff interaction (Sopa et al., 2020). Quality is “the standard of something as measured against other things of a similar kind; the degree of excellence of something” (Mustafa et al., 2020). Some other perspectives about the quality are “a degree of excellence,” “a special or distinguishing attribute,” and “something that serves to identify a subject of perception or thought in respect of which it is considered (Novitasari et al., 2020).” Quality improvement in the education sector is a strategy for creating a competitive environment in higher education by utilizing the human resources of the education management system to provide an enhanced network of services (Kooli and Abadli, 2021). To increase quality, one must listen carefully to the student’s opinions. Moreover, considering the equal importance of service quality for the given reason, it is essential to understand that recent literature differentiates the four elements of service quality in higher education in China as “competence, tangibles, responsiveness, and convenience” Carter et al. (2020). In this context, the two most significant aspects of service quality are expertise and tangibles. To provide a comprehensive customer experience, higher education institutions must focus on processes such as investigation, administrative support, advisory actions, community contribution, and teaching. When quality improvement is applied, according to Mustafa et al. (2020), business and management performance is improved, and the cost of service delivery is lowered.

Gandhi discussed the evolution and significance of quality in higher education. He believes that higher education is a tool for change and improvement and aids in the preparation of leaders. It is especially true in a country like China, where only a few reputable universities offer the necessary level of education (Mohamed Hashim et al., 2022). Similarly, it must align with current professional and market trends to make higher education more output-oriented. Likewise, the significance of merging quality management with university education was addressed by Mulay and Khanna (2020). They intended that firms use their management systems to address a variety of methods, resulting in a holistic quality improvement program.

Moreover, Quality management has two levels of focus: “one is procedures: teaching and learning and supportive procedures; and second is quality management fundamentals: customer satisfaction, administration, and employee participation” (Steppacher et al., 2021). According to Arrieta and Avolio (2020), quality assurance and management systems are crucial components of a quality improvement project. In the same way, a quality management system requires and includes employee engagement, encouragement, accountability, leadership qualities, and a student-centered approach. The diversity management of processes that technological innovations have transformed can also improve the quality management in the admission process in educational institutes (Thornton et al., 2022). Furthermore, the admission process provided by higher education includes the distribution of actual space for courses, room management, transcripts, quality management, and leadership staff interaction, institution productivity, measurement and control, certification and credential distribution, payments of contractors, and the development of foreign collaborations with innovation capabilities (Agung Nugroho et al., 2020; Prameswari et al., 2020). In higher education, giving students assistance, guidance, and counseling services has gained practical significance for providing quality education services (Arrieta and Avolio, 2020; Iqbal et al., 2021). The sensation is formed by interpersonal relationships between consumers of these facilities and the staff. In this approach, training staff members on how to satisfy customers through prompt complaint handling, and quick response to the queries raised, are critical indicators for success.

Quality control and measurements are frequently disputed and regarded as essential in higher educational institutions, considering the contemporary competition criteria that expect increased visibility and social obligation (Mulay and Khanna, 2020; Novitasari et al., 2020; Sopa et al., 2020). With several stakeholders and processes involved in the overall education service delivery process (i.e., students, faculty members, administrative staff, society, studies, the learning process, and multiple evaluation techniques), it has always been a complicated subject how to control quality implementation and its measurement (Iqbal et al., 2021). Prameswari et al. (2020) state that intake level, curricula, academic infrastructure, sector interactions, consumer experience, related infrastructure, and extracurricular activities are essential in higher education. Arrieta and Avolio (2020) and Iqbal et al. (2021) established a concept for evaluating quality management. The terms “administrative quality” and “administrative processes” match some of the authors’ response factors. These factors attract international and local students to be a part of a standard quality institute delivering all these facilities on the campus. Despite providing all these infrastructures, a good and well-thought admission process is required to meet quality management standards and satisfy prospects.

Lastly, it is indicated that engaging appropriately with institutional innovation has become a coping strategy (Alotaibi et al., 2016). Firms successfully manage institutional innovation by integrating their efficient and productive capabilities. The spectrum of innovation, which includes norms, procedures, administrative processes, and current goods and services, is one of the two aspects of innovation (Abbas, 2020). The other half is the breadth of innovation and quality management, which are critical to this article’s success and define the student’s motivation, innovation, and business results.

The pandemic discusses the challenges of face-to-face education. Virtual university programs could continue to operate, and their other universities quickly followed suit, launching online admissions and registration for their online learning programs. This modification to the enrollment process triggered a massive revolution in the educational system. However, some concern was expressed about the quality of information security for newly enrolled students’ personal information (Mohamed Hashim et al., 2022). Through a transformed admission process, institutions innovated their admission process to maintain quality education services, and they integrated artificial intelligence into their online admission process to protect individual information. Nonetheless, the institutes are vulnerable to fraudulent data extraction (Gudiño Paredes et al., 2021). In order to maintain quality management and introduce innovative capabilities in higher education, the Open University of Tanzania has implemented information communication technology to stabilize the online education process for students, such as online enrollment and online registration. Virtual student interviews were conducted to maintain the quality of the admission process, and they have innovatively strategized their systems by implementing an online application registration process for taking classes (Oreku, 2021).

According to a study Assiri et al. (2022) on the admission process of Jazan University, the administration has strategized their admission process to maintain the quality of enrolled applicants to the Saudi university. The university has innovated its enrollment process so that if a student chooses the incorrect field, they can analyze and change their study path on time. The university has designed two pre-enrollment tests for this purpose: the GAT (General Aptitude Test) and the AT (Aptitude Test) (Achievement Test). These tests help students get into programs that are a good fit for their abilities. However, these tests are insufficient to assess a degree candidate’s ability or credibility. One possible explanation is that these tests are so difficult to pass that students form inaccurate perceptions and analyses of themselves (Van Bao and Cho, 2022).

Thus, we hypothesized that;

*H1a: There is a significant effect of the admission process on innovation capability through quality management and leadership.*

*H1b: There is a significant effect of the admission process on innovation capability through quality regarding staff interaction.*

*H1c: There is a significant effect of the admission process on innovation capability through quality in institute productivity.*

*H1d: There is a significant effect of the admission process on innovation capability through quality in terms of control and measurement process.*

### Exam process and innovation capabilities in higher education through quality management

The educational sector is essential to a country’s growth and development. In this respect, education is critical to enhancing students’ educational quality and well-being and the overall country’s performance. Similarly, in many aspects of life, quality is one of the most desired characteristics (Mulay and Khanna, 2020). While, in a given time frame, HEIS is considered to be the place striving for innovative process adoption in pursuit of total creative knowledge production and dissemination mechanism (Bano and Taylor, 2015). Quality is determined by customer expectations and the value customers obtain from a service. As a result, quality is linked to an institution’s revenue and profit. Because of the positive influence that service quality has on businesses, many scholars and practitioners have spent time and effort enhancing concepts and developing accurate and adaptable measures to assess service quality.

Many scholars and practitioners have spent time and effort enhancing concepts and strategies and developing accurate and replicable measures to evaluate quality management (Chi et al., 2018; Garba and Kraemer-Mbula, 2018). Quality improvement through online testing and evaluation is another element that has gained popularity and led to new criteria for high-quality education. Modern learning management systems (LMS) are constructed with such a sophisticated system that a sophisticated investigation and examination formation is produced to raise the standard of education globally (Arafat, 2022). In the case of Chinese students, the quality of the educational process is judged based on passing percentage. This process starts at a young age; in fact, Chinese formal education stresses testing starting at 2 years, with the start of the “three-point life” in the parents’ house (Kirkpatrick and Zang, 2011; Mulay and Khanna, 2020). Due to the massive volume of everyday homework, each class has an aggregate of three or four tests for each topic, providing few leisure activities or interests. Testing is used in communities today to maintain a fair and neutral learning environment. Tests are one method of assessing the effectiveness of learning and education. There is a universal consensus in education that testing impacts the teaching process, a phenomenon known as the “academic achievement of students” (Arrieta and Avolio, 2020). Based on the circumstances of the test, this influence might be both beneficial and detrimental. The concept of effective testing is also mentioned by Engdaw (2022) as, in effect and effect, driven testing, test creation in which the impacts drive the ultimate test design decisions that the test will have on stakeholders.

Currently, the education system across china is managed by the Ministry of Education, a mostly government-run system. In the given system, exams are the baseline for measuring students’ academic performance. Likewise, as soon as pupils begin school in China, they are subjected to several exams (Qi, 2004). Furthermore, with the ever-rising need and interest of aspirants in higher education, Govt. of China is making use of several examination processes as a measure of gauging students learning outcomes and the intensity of quality of education they have received (Hill et al., 2010). Similarly, students in China take the National Higher Admittance Examination, which is the key to university entrance, after grade 12. Only top-scoring students on high-stakes tests are eligible to enroll in universities, be recruited for desirable occupations, and have access to possibilities not available to lower-scoring pupils. China’s educational system is very selective, with fewer pupils at the top of the learning curve. Students who have done well on many competitive tests, like the “NMET,” have moved up the educational ladder.

It is due to the growing demand for innovation capabilities throughout the job market in China. Previous studies have explored transitioning from effectiveness to an innovation mindset to overcome this issue. A greater understanding of how individuals can be coordinated is required to boost institutions’ creativity and productivity. Sopa et al. (2020) say that innovative process reengineering is critical for improving instructors’ performance and suggests that universities that emphasize innovation will be more creative and better able to compete in the higher education sector. The Open University of Tanzania has innovated its exam process during COVID by utilizing information and communication technology to sustain and maintain the quality of the examination process by setting the benchmark by utilizing Oral Final Examinations. This system has assisted them in conducting fair exams with no fraudulent activities (Oreku, 2021). The online examination was an innovative process, but it raised the issue of exam quality management. The use of online examination methods raised concerns about exam monitoring and control. Universities have integrated the Artificial Intelligence Program into their respective adopted online assessment tools for quality management and quality enhancement so that they can virtually and visually control their students and conduct fair assessments (Mohamed Hashim et al., 2022).

The most recent educational trend revolves around technological innovations. The innovation mechanism adopted by institutes has facilitated the continuation of virtual education but has also revealed student dissatisfaction with the innovation (Lee and Fanguy, 2022). With student dissatisfaction comes the issue of quality control measures and quality management failures that fail to assist students. During the lockdown, universities have also used Proctored exams to facilitate examinations and maintain exam quality. As students and faculty were compelled to adopt technology quickly, the Proctored exam process was designed with a strategy to balance innovation and quality management. This online integrated exam process, aided by user-friendly functions, was beneficial to both teachers and students and positively affected student satisfaction (Raman et al., 2021). This exam process also assisted faculties in providing virtual monitoring of students’ activities, which makes the education system credible and qualitatively innovative (Lee and Fanguy, 2022).

Thus, we hypothesized that;

*H2a: There is a significant effect of the exam process on innovation capability through quality management and leadership.*

*H2b: There is a significant effect of the exam process on innovation capability through quality regarding staff interaction.*

*H2c: There is a significant effect of the exam process on innovation capability through quality in institute productivity.*

*H2d: There is a significant effect of the exam process on innovation capability through quality in terms of control and measurement process.*

### Placement process and innovation capabilities in higher education through quality management

In the same way, the consequences of the placement process in higher education have been the focus of much research. The placement process has been linked to increased learning outcomes (Agung Nugroho et al., 2020), work preparedness, personality, and team abilities (Iqbal et al., 2021), graduating teachers (Schuch et al., 2018), and an increased income for trainees (Tarí and Dick, 2016). Staff happiness, enrollment, pre-professional image, and educational success all benefit from the placement process (Zhang et al., 2018; Abou-Khalil et al., 2021; Caspersen and Smeby, 2021). The placement process is emphasized as a complement to regular university instruction, not as a replacement.

There is a significant focus on the placement process and professional skills to improve educational results in higher education practice and legislation (Mustafa et al., 2020). University-based programs are often considered adequate in preparing students for a seamless transition into the workforce. Another element included in the knowledge curriculum for students in higher education is practice-based learning, which exposes students to real-world situations and develops their practical knowledge, both of which improve the quality of education (O’Leary et al., 2022). Industrial knowledge and job skills are critical components of teachers’ competitiveness growth (Zhang et al., 2018). Additionally, some countries and institutes connect apprenticeships method of learning to each concentration on the placement process and placement level and relate a consistent view of learning outcomes to a focus on the assimilation of learning during the placement process (Moore, 2014; Abou-Khalil et al., 2021; Caspersen and Smeby, 2021).

It is unclear whether emphasizing a vocational education and placement level is sufficient or a consistent curriculum design is also required. It is also suggested that simply presenting the results is adequate and that more investigation into the relationships and application of the approach is required to yield several outcomes in higher education (Stephens, 2022). The placement of education quality also depends on the caliber of knowledge and instruction provided by the instructor and the kind of student aptitude that contributes to the achievement of education quality (Manderstedt et al., 2022). Additionally, the placement of an education system with quality sustainability has many various forms that cannot be directly linked to any one set of factors or actions but can be changed periodically as a result of the ongoing learning opportunities that come with skill enhancement and development (Harrison et al., 2022).

A few essential characteristics can be used to identify the placement process with quality improvement in the education sector, including vital academic research, knowledge enhancement, development and sharing culture, curriculum development and up-gradation, student-teacher collaboration, and education management structures created to maintain quality with novelty (Abbass et al., 2022). According to Nawaz Khan et al. (2019), a focus on quality and efficiency in higher education proliferates between governments and participants. Their research uncovered some crucial aspects of higher education quality control and measurement. Specifically, the administrative staff provides an internal focus on quality for the research, as they are an essential internal user in a higher education series of placement processes. Other studies have defined an institutional system as a group of subsystems and procedures with coordinated outputs and inputs that provide value (Baber, 2021). The emphasis has shifted toward having creative teachers experience innovation capabilities, institutional operations, and behavior among all staff to have an education system focused on industrial linkage and placement of its graduates. Past studies have looked into transitioning from effectiveness to an innovation mindset to combat this problem. A greater understanding of how people are linked to boosting institutional creativity and productivity (both within and beyond) is required (Prameswari et al., 2020). Therefore there is an ever-rising need for the system-wise provision of innovation across academic institutes to ensure industrial linkage for placement purposes.

Furthermore, Mulay and Khanna (2020) say that processes should generate innovations that aid in quality management. At the same time, Garba and Kraemer-Mbula (2018) indicates that individual innovation influences the institution’s worth through its impact on profitability and financial situation. Nonetheless, Sopa et al. (2020) state that innovation is critical for increasing teachers’ output. They demonstrate that institutions prioritizing teachers’ innovation capabilities will be more efficient and compete in the global marketplace. While Innovation capability can be defined as “The process innovation is a process that a firm can deliver a better service process that helps to achieve a better performance” (Abbas, 2020). Similarly, the variables of Quality improvement through skilled teachers and creative processes for handling quality concerns in institutes were discussed by Caspersen and Smeby (2021). One of the issues, they claim, is the placement process through innovation capabilities. While the placement process plays a vital role, universities must examine their institutional framework more closely to improve quality through innovation. China’s educational institution is very selective, with fewer pupils at the top of the career ladder for the placement process.

University placement opportunities incorporate work-based learning programs that allow students to gain practical work experience by learning and practicing. Such innovative initiatives in placement programs assist students in expanding their knowledge, developing skills, and obtaining placement opportunities. Such innovative initiatives assist higher education institutions in maintaining their quality management factor (Jung, 2022). The work integrated learning system WIL was established in Vietnamese universities to provide students with placement opportunities from local to international workplace territories. WIL was initially successful but lost quality due to time constraints and a lack of interaction between teachers, students, and supervisors (Ha, 2022).

Engineering faculties at South African universities of technology have adopted the beneficial WIL system. They have transformed the process from full-time placement to a 6-month practical experience-gaining program in which students in their second or third semesters work off campus in a realistic environment (Ngonda et al., 2022). This placement process helps maintain the educational quality management of the institutes by providing students with new experiences and connecting them with relevant professional organizations. This method offers students a well-structured path to employment.

Thus, we hypothesized as follows;

*H3a: The placement process significantly affects innovation capability through quality management and leadership.*

*H3b: There is a significant effect of the placement process on innovation capability through quality regarding staff interaction.*

*H3c: The placement process significantly affects innovation capability through quality in institute productivity.*

*H3d: There is a significant effect of the placement process on innovation capability through quality in terms of control and measurement process.*

### Resource-based theory

The present study established the resource-based view theory and can be described as “a human, dynamic and social approach for strategy formulation. It is conceived and executed by a subjective, interactive process driven by human beings, based on their beliefs and judgments and actions taken within particular contexts with the common good in mind” (Iqbal et al., 2021). Moreover, according to the research, firms must deal with innovative efforts by implementing advanced HR practices to control organizational management and adjust to new information and innovation capabilities. The fast development of information technology results in several organizational advancements, particularly helping organizations update their policies (Alotaibi et al., 2016). As a result of these differences, businesses’ existence and durability are increasingly dependent on some elements, including quality management and acquiring innovation capabilities.

For this reason, knowledge management has become critical in ensuring organizational effectiveness. On the other hand, According to the Resource-based view method, institutions that use their valued resources, such as staff expertise, are more likely to succeed. In another way, “it is revealed that organizations use their employee’s knowledge as an inimitable resource to attain innovative advantages” (Douglas and Sutton, 2010; Mulay and Khanna, 2020). Additionally, quality management uses behavioral techniques and transformation to engage, inspire, and retain personnel (Tarí and Dick, 2016). Staff interaction with the perspective of resource-based view theory suggests that technological and quality advancement awareness is critical to overall firm performance (Arrieta and Avolio, 2020). Therefore, in line with the resource-based view, the given study looks into the creation of innovation capabilities through convergence mechanisms across higher education institutions from the viewpoint of quality management and leadership, staff interaction, institution productivity, and control and measurement process.

## Methodology

The proposed hypothesis was evaluated using a quantitative research technique in the current study. This method has aided in the reduction of bias. This quantitative study used a self-administered survey to collect data. Furthermore, teachers from several Chinese universities were included in the study’s population. Although University teachers were chosen for this study, it did not prove easy to collect responses from all faculty members simultaneously. As a result, the researcher employed stratified sampling. When researchers need to divide a sample into identical groups, they use this technique. As a result, for this study, teachers were divided into groups based on their universities and fields of specialization. Data collection from groups had thus become simpler. The researchers used this sampling strategy to collect data primarily from higher education teachers. The survey questionnaires were delivered to survey respondents and collected within a week. A total of 350 completed surveys were received and reviewed. The current study’s observational unit was higher education teachers.

PLS-SEM analysis was used in this study. Typically, two software tools are used to perform SEM analysis: PLS and AMOS. PLS is used to perform SEM analysis in this study. This is because PLS does not impose any restrictions on normality measurements. Unlike other SEM analysis tools, PLS is flexible for its small size (Nawaz et al., 2019). This software tool is also used in this study to make predictions about the relationships between the construct variables (Nawaz et al., 2021). Amos is used for studies in which researchers want to test theoretically assumed constructs. As a result, this tool is the best option for this study.

### Measurements

The research tool for this study was a questionnaire. The questionnaire included a section for each variable. Among the variables studied were admissions, exams, placement, higher education quality, staff interaction, measurement and control, and innovation capabilities. The scale used in the study had previously been used in similar studies. For the admissions process, a four-item scale was used (Arrieta and Avolio, 2020; Mulay and Khanna, 2020). Questions included in this scale are “Thorough selection process that ensures the students’ academic level” and “Thorough selection process that ensures the students’ performance.”

A 4-item scale of the exam process was adopted by Arrieta and Avolio (2020) and Mulay and Khanna (2020). Questions included in this scale are “Update class slides” and “Effectively schedule midterm and final exams.”

A 3-item scale of the placement process was adopted by Lin (1998) and Mulay and Khanna (2020). Questions included in this scale are “Being placed at university sites that are closest to my housing resources” and “Being placed at clinical sites that are closest to my family.”

A 4-item scale in terms of quality management and leadership was adopted by Mulay and Khanna (2020). The question included in this scale is “A written policy statement or manual that defines a quality program is readily available at the university.”

A 4-item scale of quality in terms of staff interaction was adopted by Lin (1998) and Mulay and Khanna (2020). The question included in this scale is, “Appropriate procedures are in place to assure those customer complaints are responded to promptly.”

A 4-item scale of quality in terms of institute productivity was adopted by Lin (1998) and Mulay and Khanna (2020). The question included in this scale is “Effort is made to get opinions and suggestions from people at the university.”

A 5-item scale of quality in terms of measurement and control was adopted by Lin (1998) and Mulay and Khanna (2020). The question included in this scale is “Top management follows up suggestions for improvement.”

A 5-item scale of quality in terms of innovation capabilities was adopted by Cheng and Chen (2013). The question included in this scale is “Identifying new product opportunities.” The results were collected by a “7-point Likert scale ranging from 1 = Strongly Disagree to 7 = strongly agree.”

## Findings

### Demographics

A total of 350 completed surveys were received and examined. In the Table 1, gender description, there were 63% males and 37% females. According to age differences, 35–45 years was 51%, above 45 years was 26%, and 25–35 years was 23%. Regarding qualification or education, there were 57% Ph.D. and 43% master’s degree holders. According to their hiring or positioning, associate professors were 43%, assistant professors 37%, and lecturers 20%.

**TABLE 1:** Demographics.

Demographics	Description	No of responses	Percentage
**Gender**			
	Male	220	63%
	Female	130	37%
**Age (years)**			
	25–35	80	23%
	35–45	180	51%
	Above 45	90	26%
**Qualification**			
	Masters	150	43%
	Ph.D. or others	200	57%
**Positions**			
	Assistance professors	130	37%
	Associate professors	150	43%
	Lecturer	70	20%

### Measurement model

**FIGURE 2:**
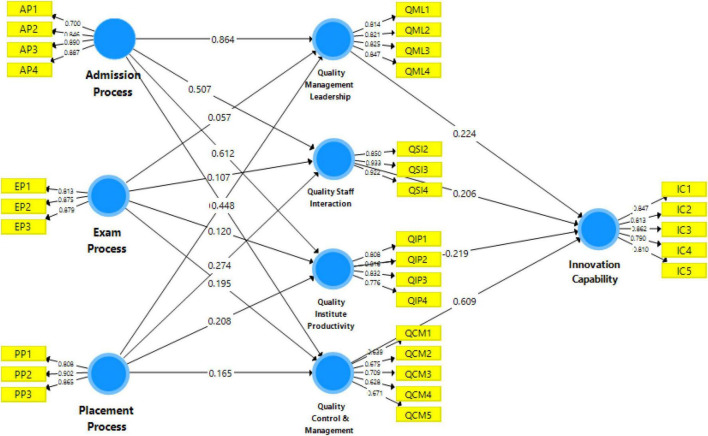
Measurement algorithm model.

The measurement model is “the relationship between the observed or indicators and the latent variables” (Sarstedt et al., 2020). Figure 2 shows the measurement model algorithm in this study, which shows dependent and independent variables data.

Furthermore, the measurement model was used to calculate “Cronbach’s alpha (CA) and composite reliability (CR)” to examine the coherence of the measurements. “CA and CR values greater than 0.7” were found in all investigation items, indicating that they met the reliability criterion (Hair et al., 2020). Factor loading levels have been classified into three categories by Lamber et al. (1991), namely “unattractive (value 0.3), acceptable (value > 0.5), and extremely desirable (value > 0.7)” (Sarstedt et al., 2020). As a result, each item contributed a great deal to the current study. The value of CA ranges from “0 to 1, and it is also divided into three categories; fair reliability (value of 0.6), satisfactory reliability (value from 0.6 to 0.7), and highly satisfactory reliability (value from 0.7 to 0.9) (Iuliano et al., 2018).”

In the Table 2, the values of CA for each variable were 0.784–0.886, which was indeed accepted. The values of CR for each variable were 0.709–0.888, which was highly satisfactory. Moreover, the value of AVE variables was above 0.50, and all were accepted. According to Fornell and Larcker (1981), discriminant validity is “the amount to which a given latent variable differs from other latent variables.” It was calculated by looking at the correlation between the latent variable item and the actual number of AVEs. When establishing discriminant validity (see Table 3), it recommends using latent components with a value of 0.50 or higher (Yingfei et al., 2021). Discriminant validity explains whether or not one construct is distinct from another. Likewise, “the square root of every variable’s (AVE) must be greater than the highest relationship of the construct with the other latent variable to assess the discriminant validity of the construct using the Fornell and Larker Criterion” (Avotra et al., 2021). The Table 2 demonstrates that the value at the head of each column is greater than the amount below it, showing that the variables have discriminant validity.

**TABLE 2:** Composite reliability, Cronbach’s alpha, and AVE values.

Constructs	Cronbach’s alpha	Composite reliability	AVE
Admission process	0.852	0.901	0.696
Exam process	0.818	0.892	0.733
Innovation capability	0.882	0.914	0.68
Placement process	0.824	0.894	0.738
Quality control and management	0.784	0.845	0.664
Quality institute productivity	0.833	0.883	0.653
Quality management leadership	0.846	0.896	0.683
Quality staff interaction	0.886	0.929	0.814

CR, composite reliability; AVE, average variance extracted; CA, Cronbach’s alpha.

**TABLE 3:** Discriminant validity.

Constructs	AP	EP	IC	PP	QCM	QIP	QML	QSI
Admission process	**0.834**							
Exam process	0.382	**0.856**						
Innovation capability	0.512	0.311	**0.825**					
Placement process	0.323	0.438	0.297	**0.859**				
Quality control and management	0.576	0.438	0.417	0.396	**0.665**			
Quality institute productivity	0.674	0.444	0.466	0.458	0.432	**0.808**		
Quality management leadership	0.615	0.421	0.573	0.384	0.361	0.224	**0.827**	
Quality staff interaction	0.636	0.421	0.586	0.485	0.412	0.397	0.219	**0.902**

AP, admission process; PP, placement process; EP, exam process; QIP, quality institute productivity; QSI, quality staff interaction; IC, innovation capability; QML, quality management and leadership. Bold values shows the relationship between the variable and its significance value.

The value of *R*^2^ ranges from zero to one. Moreover, Sarstedt et al. (2020) recommended that the *R*^2^ of “0.13 is considered weak,” “0.33 is moderate,” and “0.67 is considered as strong.” *R* Square “explains the variance in the endogenous variable explained by the exogenous variable.” In the below table, the *R* square value of innovation capability was 0.572, and quality institute productivity was 0.591. It considers moderate; the *R* square value of quality management and leadership was 0.839. It considers vital, and the *R* square value of quality staff interaction was 0.500. It is also considered moderate, and the *R* square value of quality control and management was 0.409, which is considered moderate as shown in Table 4.

**TABLE 4:** Assessment of *R* square.

Constructs	*R* square
Innovation capability	0.572
Quality control and management	0.409
Quality institute productivity	0.591
Quality management leadership	0.839
Quality staff interaction	0.500

### Structural model

**FIGURE 3:**
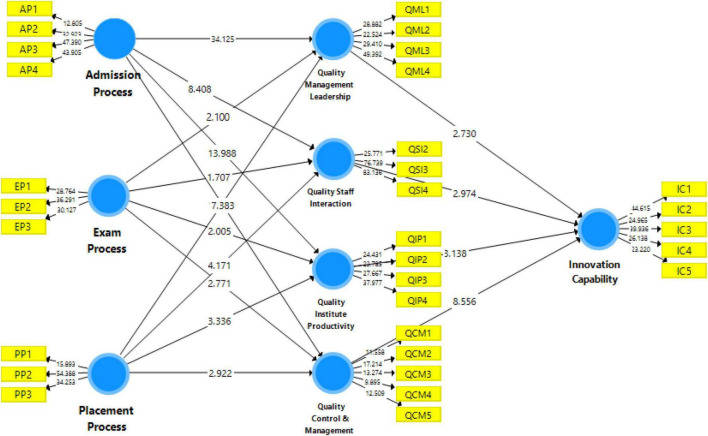
Structural model bootstrapping.

The structural model is “a multivariate statistical technique that allows researchers to estimate and test causal relationships” (Xiaolong et al., 2021; Nawaz et al., 2022). Figure 3 shows structural model bootstrapping in this study, which shows dependent and independent variables data.

The proposed model for the study uses a structural model to stress the interconnectedness of the relationships. The structural model in PLS looks at the direct relationship between the offered hypotheses and their *t*-values and regression coefficients; an indirect effect is the same as a standardized beta value in regression analysis (Hair et al., 2019). The *t*-values and beta values of the regression coefficients are used to determine significance; *t*-values more than “1.64” are statistically significant and are then used to make conclusions about the suggested hypothesis (Sarstedt et al., 2020). The model’s two primary purposes are to examine direct linkages and verify projected interactions between components using a structural model. All hypotheses were accepted as the *p*-value is less than 0.05 and the *t*-value above 1.64 for one-tailed, also known as directional hypotheses (see Table 5).

**TABLE 5:** Direct hypothesis testing.

Direct hypotheses	Beta	Standard deviation	*T* statistics	*P*-values	Decision
Admission process → quality control and management	0.448	0.061	7.383	0.000	Supported
Admission process → quality institute productivity	0.612	0.044	13.988	0.000	Supported
Admission process → quality management leadership	0.864	0.025	34.125	0.000	Supported
Admission process → quality staff interaction	0.507	0.060	8.408	0.000	Supported
Exam process → quality control and management	0.195	0.070	2.771	0.004	Supported
Exam process → quality institute productivity	0.120	0.060	2.005	0.025	Supported
Exam process → quality management leadership	0.057	0.027	2.100	0.020	Supported
Exam process → quality staff interaction	0.107	0.063	1.707	0.046	Supported
Placement process → quality control and management	0.165	0.057	2.922	0.002	Supported
Placement process → quality institute productivity	0.208	0.062	3.336	0.001	Supported
Placement process → quality management leadership	0.075	0.038	1.997	0.025	Supported
Placement process → quality staff interaction	0.274	0.066	4.171	0.000	Supported
Quality control and management → innovation capability	0.609	0.071	8.556	0.000	Supported
Quality institute productivity → innovation capability	0.219	0.070	3.138	0.001	Supported
Quality management leadership → innovation capability	0.224	0.082	2.730	0.004	Supported
Quality staff interaction → innovation capability	0.206	0.069	2.974	0.002	Supported

AP, admission process; PP, placement process; EP, exam process; QIP, quality institute productivity; QSI, quality staff interaction; IC, innovation capability; QML, quality management and leadership.

Moreover, the indirect hypotheses of the study are also supported. The results also show the indirect effects that exposed the mediation analysis. The results indicated that the study framework’s total, direct and indirect effects are accepted. These relationships are shown in Table 6.

**TABLE 6:** Mediation hypothesis testing.

Mediation hypotheses	Beta	*SD*	*T* stats	*P*-values	5.00%	95.00%	Results
Admission process → quality control and management → innovation capability	0.273	0.050	5.419	0.001	0.244	0.339	Mediation
Exam process → quality control and management → innovation capability	0.119	0.047	2.513	0.023	0.060	0.140	Mediation
Placement process → quality control and management → innovation capability	0.101	0.026	3.898	0.004	0.062	0.111	Mediation
Admission process → quality institute productivity → innovation capability	0.134	0.036	3.757	0.005	0.177	0.118	Mediation
Exam process → quality institute productivity → innovation capability	0.026	0.005	5.732	0.001	0.027	0.026	Mediation
Placement process → quality institute productivity → innovation capability	0.046	0.021	2.202	0.035	0.078	0.039	Mediation
Admission process → quality management leadership → innovation capability	0.194	0.054	3.566	0.006	0.107	0.211	Mediation
Exam process → quality management leadership → innovation capability	0.013	0.006	2.034	0.044	0.007	0.013	Mediation
Placement process → quality management leadership → innovation capability	0.017	0.007	2.342	0.029	0.011	0.013	Mediation
Admission process → quality staff interaction → innovation capability	0.104	0.040	2.633	0.019	0.054	0.133	Mediation
Exam process → quality staff interaction → innovation capability	0.022	0.010	2.123	0.039	0.014	0.022	Mediation
Placement process → quality staff interaction → innovation capability	0.056	0.020	2.872	0.014	0.031	0.066	Mediation

AP, admission process; PP, placement process; EP, exam process; QIP, quality institute productivity; QSI, quality staff interaction; IC, innovation capability; QML, quality management and leadership.

## Discussion

The current study focuses on innovation capabilities in higher education convergence trends from the standpoint of quality management. This study developed a framework for connecting stakeholder interests with the quality of service delivered. As a result, managers can actively seek to improve educational quality. Most universities, including higher education institutions, are implementing quality-control measures that take a holistic approach to the comprehensive processes of the institutions. The study’s findings will help organizations identify the factors that influence the quality of their outputs. This study was founded on resource-based theory regarding admission, exam, and placement. It implies that the admissions process significantly impacts the overall quality of management through the institution’s innovation capabilities because the admissions process is a precise and accurate predictor of quality in higher education. The previous study by Mulay and Khanna (2020), indicates similar perceptions by stating that the admission process and quality management has a significant impact. Through innovation capabilities, admissions are more strongly related to the “quality of staff interaction.” On the other hand, staff interaction quality has a significant relationship with the “admission process” (Mulay and Khanna, 2020).

Furthermore, admissions have the most significant impact on quality institute productivity through innovation capabilities. The university will be more likely to select top students and achieve better results if there is a high-quality admissions process (Mulay and Khanna, 2020). The standards for quality improvement in an institution’s admission process are also influenced by student expectations, constantly evolving and changing over time. Higher education institutions should become proactive and get top students on board to recruit diversified students, which will bring more transformation to the quality management process of the educational institutes (Thornton et al., 2022). Other suggested quality admission process indicators include course distribution, room and transcript process management, staff interaction, institutional policies, procedures and control, and collaboration with international institutions, which all combine to form innovative capacities that provide quality management standards (Agung Nugroho et al., 2020; Prameswari et al., 2020). Student counseling, assistance, and guidance in admission are a few other aspects (Arrieta and Avolio, 2020; Iqbal et al., 2021). Finally, the findings show that the admissions process significantly impacts quality control and measurement via innovation capabilities. Overall, the admissions process impacts quality through innovation more than other variables.

On the other hand, the researchers’ investigation develops deeper into some specific administrative processes, which they did not do in their previous research (Mulay and Khanna, 2020; Steppacher et al., 2021). The Exam process has a much smaller impact on the institution’s management and leadership quality than the admission process. As evidenced by the literature, the desired improvements in the examination process can be achieved through staff interaction. The measure of staff interaction demonstrates the examination process’s ability to improve (Mulay and Khanna, 2020). The exam process also demonstrates the efficacy of the learning and education process. Teaching methods universally assess education quality, and teachers are further assessed through continuous testing and examination (Arrieta and Avolio, 2020). This impact can be functional and dysfunctional, depending on the situation of the test. It shows that the hypothesis has been accepted, but the impact obtained is less than perceived by literature studies. The exam process also demonstrates the efficacy of the learning and education process. Teaching methods universally assess education quality, and teachers are further assessed through continuous testing and examination (Arrieta and Avolio, 2020). This impact can be functional and dysfunctional, depending on the situation of the test. Today, institutions’ innovation processes are incomplete without an effective examination process, as it indicates teachers’ quality teaching methods and innovative capabilities for education quality management in universities (Sopa et al., 2020). The standards for innovative capabilities are also indicated by technology-based education mechanisms, such as the LMS system, designed to raise sophisticated standards of quality education (Arafat, 2022). While examination processes significantly impact quality institution productivity through innovation capabilities, the university will have a better chance of selecting top students and achieving better results if the exam process is of high quality (Mulay and Khanna, 2020). Finally, the findings show that the exam process significantly impacts quality control and measurement via innovation capabilities. The examination process has a significant impact on the quality of the institution.

Furthermore, the placement process has a minor impact on the quality of management and leadership through the institution’s innovation capabilities than the admission process. There is a strong link between the “placement process and quality management” (Mulay and Khanna, 2020). The placement process focuses on improving educational quality through improved professional skills (Mustafa et al., 2020). The placement programs offered by universities help in the skill transitioning of students. However, it is still unclear whether new curriculum reform models should be introduced or whether vocational education is an excellent addition to the placement process (Stephens, 2022). Aside from curriculum design, another factor that influences a higher education institute’s quality is the caliber of its instructors and students (Manderstedt et al., 2022). Through innovation capabilities, placement is more accurately related to the quality of staff interaction. The quality of staff interaction significantly impacts the placement process (Mulay and Khanna, 2020). The placement has the most significant impact on quality institute performance due to its innovative capabilities. Finally, the findings indicate that the placement process significantly impacts “quality control and measurement” via innovation capabilities. Furthermore, the current study’s researcher discovered that the placement process has a significant relationship with quality implementation in higher education (Garba and Kraemer-Mbula, 2018; Mulay and Khanna, 2020). Because all factors are interconnected, any inappropriate or incorrect activity in the procedures will decline the institution’s quality, whereas good management and quality-oriented academic processes will only result in higher performance.

## Implications of study

### Practical implications of study

This study aimed to make theoretical and practical advances in understanding. Furthermore, this research provides policymakers, practitioners, and higher education administrators with the helpful information in various ways. For starters, the admissions, exam, and placement processes significantly impact quality management via innovation capabilities. As a result, the institution’s “admissions, exam, and placement process” activities must be improved and enhanced to ensure high quality in these areas. Second, as a proactive strategy, the exam, placement, and admission processes should be evaluated and improved regularly. The referred changes have enforced all of these process changes, indicating flaws and areas for improvement. As a result, the findings of this study must be communicated to educational institutions in order for them to transform and become proactive in enhancing their innovative capabilities and maintaining the quality of their educational standards.

### Theoretical implications of study

This study provides a solution for educational administrators and educators. Many process innovative ideas and solutions implemented by other universities are discussed in this study’s literature. The educator can study and research them before strategizing their implications. This study adds to the body of literature by providing learners and teachers with suggestions and solutions for tracking their own and students’ educational and professional career paths. Students can use these indicators to check the quality management and process innovation used by the institutes when applying for admission to higher education institutes.

## Limitations and future study

Even though the study meets its objectives, some limitations must be noted before extrapolating its findings. The current study was conducted among university teachers. As a result, extrapolating study findings to other industries may be difficult. The study sample is restricted to Chinese institutions, and data was gathered from various universities. Future research can test this study’s conceptual framework in other countries, allowing the findings to be applied more broadly. Although the data was collected in a cross-sectional format, future researchers may use a longitudinal study design to determine causation more accurately. Furthermore, future research could put the proposed framework to the test in the context of private universities, comparing results from public and private institutions. The current study only examines teachers’ ability to innovate in higher education; the same can be said for managers in other industries.

## Conclusion

This research aimed to develop a framework for evaluating quality management in higher education institutions. The current study looked at the impact of innovation capabilities on higher education convergence trends from the perspective of quality management in Chinese universities. The Resource-Based Theory was applied to this study, and the quality improvement factors obtained from the study show that the admission process is an influencing factor for quality standards in education. The findings concluded that for admission increasing innovative capabilities for the admission process, good leadership is accompanied by a good quality management process. Leadership and quality management aid in the institution’s innovative capabilities, and with this process comes to an improved admission process. Other significant elements for developing an improved admission process that has been hypothesized include staff interaction for developing innovative education capabilities, institutional productivity, and the institutional control and measurement process, all of which combine to form a maintained improved quality for developing an improved admission process. The study’s findings also show that the exam process significantly correlates with innovative capabilities. These innovative capabilities for developing a solid exam system are accompanied by institutional quality management, leadership qualities, institutional productivity capacity, and the institutional control and measurement process.

The placement process has been discovered to impact the higher education institute’s ability to innovate, which is further accompanied by leadership, quality management, institute productivity, and the institutional control and measurement process. All three perceived variables, admission process, exam process, and placement process, are found to positively impact innovative capabilities through the moderators’ direct and indirect effects; leadership, quality management, institutional productivity, and the institutional control and measurement process. Aside from these direct influencing variables, the study discovered that leadership, quality management, institutional productivity, and the institutional control and measurement process directly impacted higher education institutes’ innovative capability. So, from the above findings, we also concluded that in Chinese higher education institutes, admission, exam, placement, and quality implementation significantly impact innovation capabilities in Chinese universities. The study’s findings have assisted institutions in determining which components need to be prioritized to improve quality. This study will assist institutions in identifying and focusing on actions to improve education quality through innovation capabilities. The study focuses on the relationship between quality services and higher education innovation capabilities and the consequences of those processes.

## Data availability statement

The original contributions presented in this study are included in the article/supplementary material, further inquiries can be directed to the corresponding author.

## Ethics statement

The studies involving human participants were reviewed and approved by Changshu Institute of Technology, China. The patients/participants provided their written informed consent to participate in this study. The study was conducted in accordance with the Declaration of Helsinki.

## Author contributions

YG: conceptualization and data collection. JW: writing the draft. Both authors agreed to the submitted version of manuscript.
